# Successful treatment of cutaneous small-vessel vasculitis with upadacitinib: a Case Report

**DOI:** 10.3389/fmed.2026.1804245

**Published:** 2026-04-14

**Authors:** Xingmo Li, Zhuochen Wu, Xin Zhao, Lijuan Liu, Huichao Li, Guoqiang Zhang, Qing Zhu

**Affiliations:** 1 Department of Dermatology, The First Hospital of Hebei Medical University, Shijiazhuang, Hebei, China; 2 Subcenter of National Clinical Research Center for Skin and Immune Diseases, Shijiazhuang, Hebei, China; 3 Hebei Provincial Innovation Center of Dermatology and Medical Cosmetology Technology, Shijiazhuang, Hebei, China

**Keywords:** cutaneous small-vessel vasculitis, JAK inhibitor, mechanism, treatment, upadacitinib

## Abstract

Cutaneous small-vessel vasculitis (CSVV) is a rare skin vasculitis that mainly affects small blood vessels and is often caused by certain drugs or diseases. When the condition is severe, skin ulcers may occur, accompanied by severe pain, which can affect daily life. This article reports a case in which the patient had a poor response to conventional corticosteroid therapy, with the condition continuing to deteriorate. Following 3 months of combination therapy with oral upadacitinib, the skin lesions nearly healed completely. This provides a new perspective for the treatment of CSVV and offers clinicians an additional treatment option.

## Introduction

Cutaneous small-vessel vasculitis (CSVV) is an inflammatory disorder that predominantly targets the small vessels, specifically referring to small-vessel vasculitis confined to the skin (1). With an estimated annual incidence of 4.5 cases per million population, CSVV is clinically uncommon; it may arise at any age but shows a predilection for adults and for females (2). In approximately half of cases no precipitating factor is identified, although the remainder are triggered by drugs or underlying disease. Implicated medications include various antibiotics, anti-arrhythmic agents such as amiodarone (3), anticonvulsants (phenytoin, valproate), allopurinol and non-steroidal anti-inflammatory drugs. Recent report has described onset after administration of biological agent therapy (4). And there was a patient who developed the same disease after receiving the COVID-19 vaccine (5). Beyond pharmacological triggers, CSVV may be associated with hematological malignancies (lymphoma, leukemia), autoimmune disorders, or infections caused by Staphylococcus aureus, Chlamydia, Neisseria species or Human Immunodeficiency Virus (HIV) (6).

The initial presentation of CSVV consists of red to pink macules and papules. These lesions progress to form the classic, fully developed signs of the disease: non-blanching petechiae and palpable purpura. The tendency for these purpuric lesions to coalesce can lead to ulceration. While less frequently observed, the clinical spectrum may also encompass urticarial plaques, hemorrhagic bullae, small vesicles, pustules, or lesions resembling erythema multiforme. The skin lesions typically present with a symmetrical distribution, most commonly affecting areas below the waist, gravity-dependent regions, or sites of constrictive clothing. While the majority of cases are asymptomatic, some patients may experience accompanying symptoms such as itching, a burning sensation, or pain. The presence of any additional systemic symptoms is contingent upon the specific underlying etiology of the vasculitis (2).

The initial treatment for CSVV typically involves a short course of prednisone at 0.5–1 mg/kg/day until no new lesions appear, followed by a rapid taper over 3–6 weeks (7). For severe or recurrent CSVV, there is currently no standardized therapeutic regimen, and the clinical evidence guiding treatment remains limited (8). Corticosteroids, non-steroidal anti-inflammatory drugs (NSAIDs), colchicine, azathioprine (9), cyclophosphamide, antihistamines, antimalarials and hydroxychloroquine have each been reported to induce remission in recurrent CSVV. Therapeutic success with mycophenolate mofetil has been documented only in isolated case reports (10). Cyclophosphamide and ciclosporin are usually reserved for refractory, severe, or secondary forms of CSVV that have failed conventional therapies. For rare, life-threatening cases, rituximab, infliximab, or intravenous immunoglobulin may be considered as alternative options. In the present case, the response to corticosteroids was inadequate, and the patient declined treatment with alkylating immunosuppressants such as cyclophosphamide. Considering that upadacitinib, a selective JAK inhibitor, blocks multiple inflammatory pathways and has demonstrated significant efficacy in the clinical treatment of atopic dermatitis, we opted to trial upadacitinib in this patient who had responded poorly to conventional corticosteroid therapy. After three months of treatment, the ulcers on both lower extremities showed near-complete healing, indicating a favorable therapeutic outcome.

## Case report

**Figure 1:**
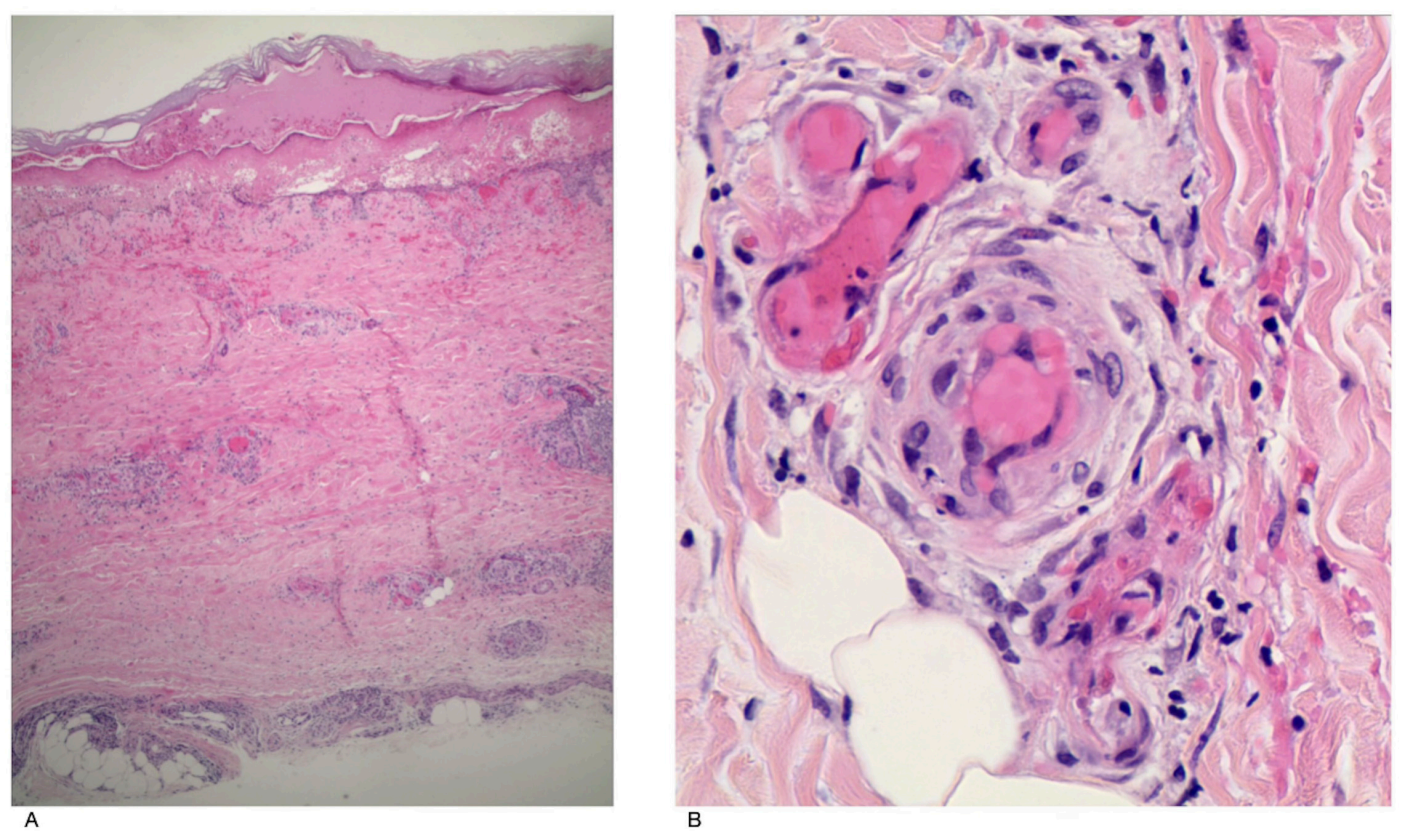
[**(A)** H&E, × 40; **(B)** H&E, × 100]. Histopathology shows epidermal hyperkeratosis, irregular hyperplasia of the spinous layer, localized ulceration with the formation of red serous crusts, unclear boundary between the epidermis and dermis, fibrinoid degeneration and thrombosis of blood vessel walls in the dermis and fat lobules. Lymphocytes, histiocytes, and eosinophils are the main inflammatory cells infiltrating around the blood vessels, with visible nuclear dust and a large number of extravasated red blood cells.

A 22-year-old male basketball player, measuring 176 cm in height and weighing 75 kg, presented to our dermatology outpatient clinic with a 15-day history of painful skin eruptions on both lower legs. The rash appeared on both calves half a month prior without any identifiable precipitating factors and was accompanied by ulceration, blister formation, and local pain. The patient had sought treatment at an external hospital, where a topical traditional Chinese medicine preparation (specific composition unknown) was applied, with poor therapeutic effect. AS the skin lesions progressively worsened, he presented to our department for further evaluation. The patient was previously healthy, with no significant past medical history. He denied a history of smoking, alcohol consumption, hepatitis, tuberculosis, thromboembolic diseases, or malignancies. Furthermore, there was no family history of similar dermatological conditions. Dermatological examination revealed the following: On the left dorsum of the foot and ankle, there were patchy erythematous lesions with relatively well-defined borders. Some lesions showed central ulceration covered with yellowish or dark-red crusts, and the ulcers margins were surrounded by erythematous halos and blisters of varying sizes. On the right dorsum of the foot and ankle, coin-sized erythematous patches with poorly defined borders were observed. Some of these lesions also exhibited central ulceration with dark-yellow to dark-red crusts. A comprehensive laboratory and imaging workup was performed, including a complete blood count, urinalysis, biochemical profile, antinuclear antibody screen, ANCA, complement, immunoglobulins, cryoglobulins negative, coagulation studies, tests for HIV, syphilis, hepatitis B and C viruses, interferon-gamma release assay (IGRA), chest computed tomography (CT), and bilateral lower extremity arterial and venous Doppler ultrasonography. All results were within normal limits or showed no significant abnormalities. The erythrocyte sedimentation rate was 12 mm/h, and the C-reactive protein was 2.95 mg/L, both within the normal range. IL-6 was 265 pg/ml, and IL-4 was 234 pg/mL. Subsequently, the patient underwent histopathological examination (Figure 1). The findings were consistent with a diagnosis of CSVV. Due to limitations in laboratory capabilities, a direct immunofluorescence test was not performed. Based on the patient's normal ANCA and IgA test results, IgA vasculitis and ANCA-associated vasculitis were ruled out. Given the patient's normal infection markers and the absence of oral medication prior to the onset of the rash, infection-triggered vasculitis and drug-induced vasculitis are temporarily considered unlikely. Based on the negative cryoglobulin test and normal complement C3 levels, cryoglobulinemic vasculitis was also excluded.

**Figure 2:**
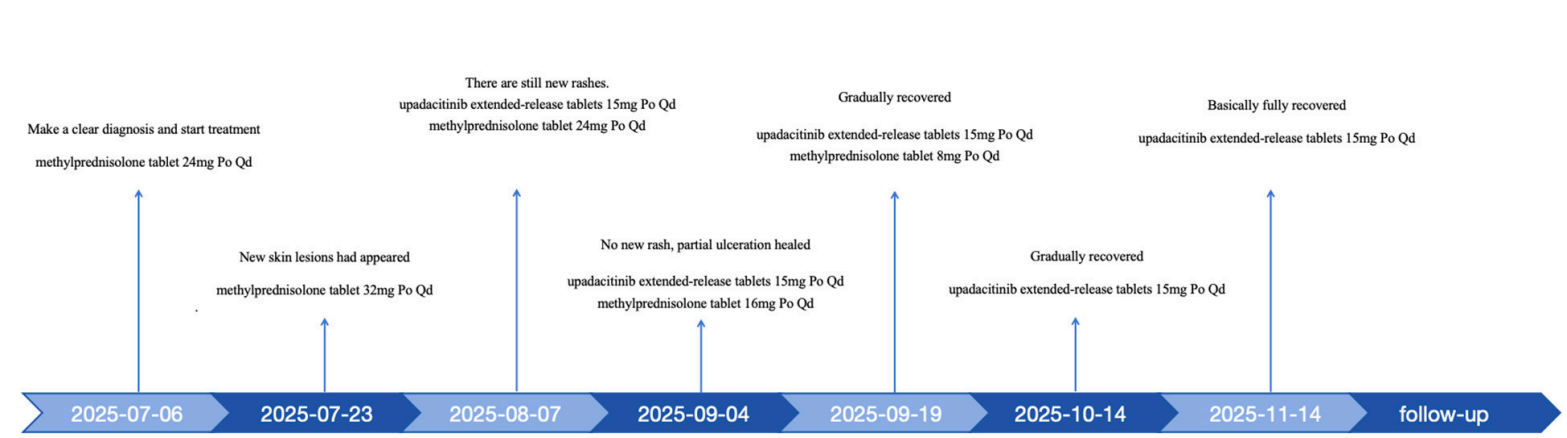
The timeline of the course of disease.

**Figure 3:**
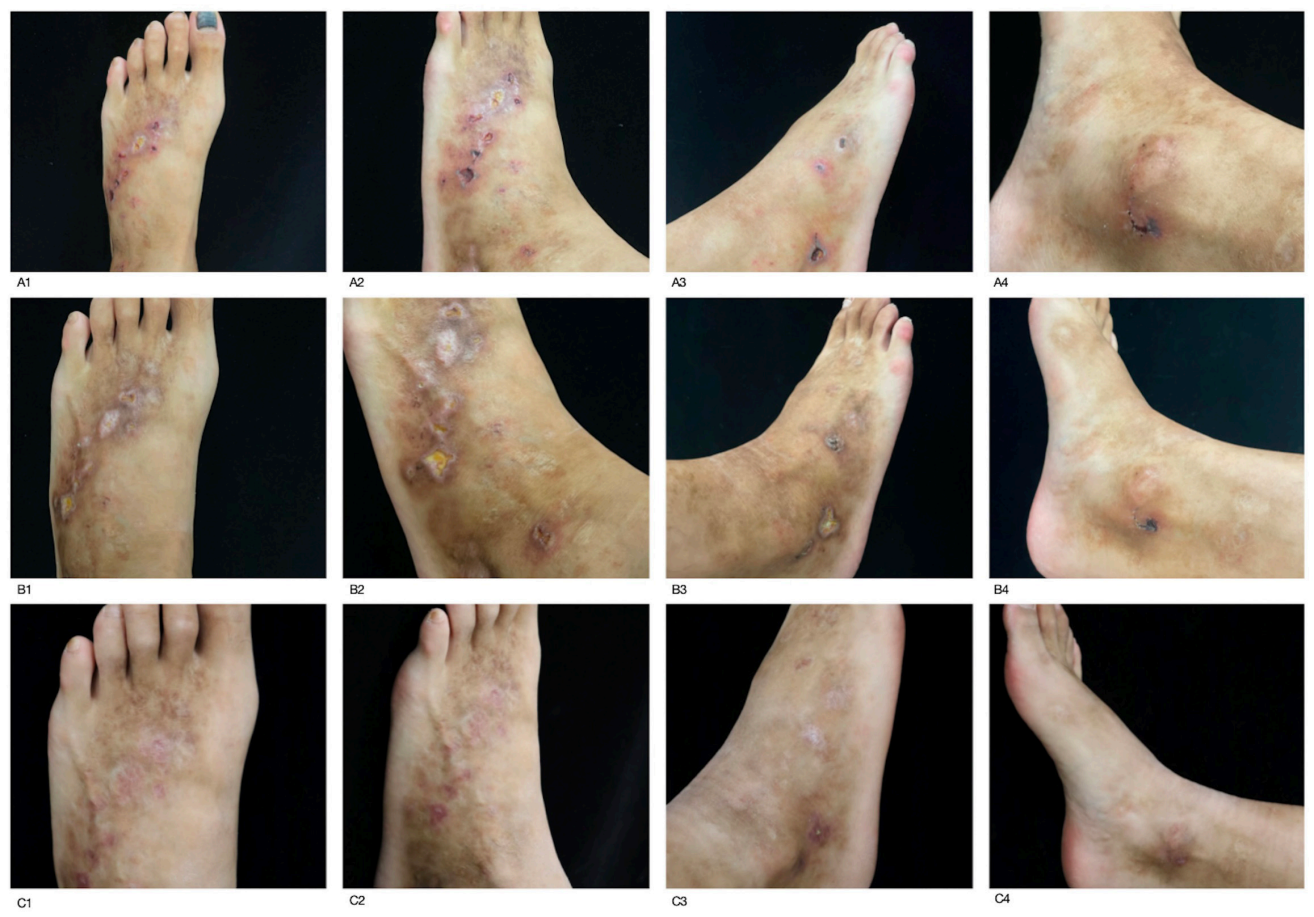
Treatment follow-up photos A1–A4: 1 month of traditional corticosteroid therapy was ineffective (2025-08-07). 13lesions noted on bilateral feet. B1–B4: Treatment with upatinib sustained-release tablets for about one month (2025-09-04). 11 lesions noted on bilateral feet. C1–C4: Treatment with upatinib sustained-release tablets for about three month (2025-11-14). 1 lesions noted on bilateral feet.

The initial treatment regimen for the patient was taking methylprednisolone 24 mg once a day, calcium dobesilate capsules 0.5 g twice a day, Xinhuang Tablets 0.96 g three times a day, compound glycyrrhizin tablets two tablets three times a day, and apply an appropriate amount of halometasone cream to the lesions twice a day. After approximately 2 weeks of therapy, re-assessment revealed that the disease activity was uncontrolled, with the number of skin lesions continuing to increase and the affected area expanding. The daily oral dose of methylprednisolone was then increased to 32 mg, and topical human epidermal growth factor gel was applied to the affected area twice a day. The doses of calcium dobesilate capsules, Xinhuang Tablets, and compound glycyrrhizin tablets remained unchanged, and the treatment continued for another half month. When the patient returned for a follow-up visit, there was still no significant improvement in the lesions, which had already affected his quality of life. Considering that after 4 weeks of full-dose methylprednisolone treatment, the patient's response was poor and the skin lesions continued to gradually worsen. After discussing with the patient and exclusion of contraindications, we decided to initiate therapy with the JAK inhibitor upadacitinib. The regimen was adjusted to take upadacitinib sustained-release tablets 15 mg once a day and methylprednisolone 24 mg everyday, while the other medications were continued at their original doses. After 1month of this treatment, the follow-up assessment indicated no new rashes and healing of some ulcerated areas. After this follow-up visit, the treatment plan was adjusted to taking upadacitinib sustained-release tablets 15 mg and methylprednisolone 16 mg everyday and continued to take compound glycyrrhizin tablets, smeared human epidermal growth factor gel. After another 2 weeks of treatment according to this plan, no new rashes had emerged. The ulcerated surfaces had healed, with some areas having formed scabs or scars. The dose of oral methylprednisolone was then reduced to 8 mg everyday, with the rest of the treatment plan unchange. After more than 20 days of continued therapy, the patient's condition was stable upon re-evaluation, and the skin rash had improved compared to before. We decided to discontinue all other oral medications, maintaining only oral upadacitinib and topical human epidermal growth factor gel. 1 month later, the patient's rash had essentially resolved (Figure 2). The treatment was streamlined to only oral upadacitinib, and the patient continues to be under follow-up (Figure 3). The cumulative dose of methylprednisolone throughout the treatment course was 2016 mg. At the 12th week of treatment, the patient underwent a comprehensive re-examination, and the results showed no significant abnormalities, allowing for ongoing oral upadacitinib treatment. We are still conducting ongoing telephone follow-ups. The patient discontinued the medication at the 24th week of treatment, and there has been no recurrence of the condition to date.

## Discussion

The precise pathogenesis of CSVV remains incompletely understood. Current evidence suggested that the core mechanism involves the deposition of immune complexes within the walls of superficial dermal small vessels. This deposition activates the complement system, generating anaphylatoxins such as C3a and C5a, which recruit and activate neutrophils. The activated neutrophils subsequently release proteolytic enzymes, reactive oxygen species (ROS), and form neutrophil extracellular traps (NETs), ultimately leading to fibrinoid necrosis of the vessel wall and the characteristic leukocytoclasis (11). This inflammatory cascade is accompanied by the release of various pro-inflammatory cytokines (e.g., IL-1, IL-4, IL-6, IL-8, TNF-α, IFN-γ) (12, 13). These cytokines are known activators of the JAK/STAT signaling pathway (14). Therefore, the JAK/STAT pathway represents a key downstream signaling hub in the inflammatory network that drives neutrophil-mediated vessel damage in CSVV.

The JAK/STAT signaling pathway comprises three core components: a tyrosine kinase-associated receptor, Janus kinases (JAK1, JAK2, JAK3, and TYK2), and signal transducers and activators of transcription (STAT1 through STAT6). Cytokines and their corresponding receptors serve as the primary activators of this pathway. Activation occurs when specific cytokines, growth factors, or ligands bind to their cognate receptors, inducing receptor dimerization and phosphorylation, which in turn initiates JAK activation. The activated JAKs then phosphorylate tyrosine residues on downstream target proteins, leading to the recruitment and subsequent phosphorylation of STAT transcription factors. Following phosphorylation, STAT proteins form homo- or heterodimers, translocate into the nucleus, and bind to specific regulatory sequences within target genes. This intricate cascade ultimately regulates the transcription of downstream genes that govern critical cellular processes such as proliferation, differentiation, and apoptosis (15).

JAK inhibitors exert anti-inflammatory and immunosuppressive effects by blocking the signaling of multiple inflammatory cytokines. Given that JAK2 inhibition may theoretically lead to neutropenia, anemia, and alterations in platelet counts, selective JAK inhibitors are considered to potentially offer a more favorable efficacy-to-safety profile compared to pan-JAK inhibitors (16). Zhu et al. (8) reported a case of refractory CSVV treated with tofacitinib. The patient received tofacitinib 5 mg twice daily in combination with prednisone 25 mg once daily. After 2 months of therapy, complete resolution of cutaneous ulcers was achieved. The patient remained in remission even after gradual tapering and discontinuation of the medications. A study by Jinlu Ma et al. (17) described three patients with CSVV who were either refractory to conventional therapies or corticosteroid-dependent. All three patients achieved significant and sustained clinical improvement following treatment with the JAK inhibitor tofacitinib, which also enabled the successful reduction or complete withdrawal of glucocorticoids. These findings suggest that JAK inhibitors represent a novel and promising therapeutic direction for patients with CSVV who do not respond to traditional treatments.

The scarcity of robust clinical studies directly comparing the efficacy and safety profiles of different JAK inhibitors makes it unclear whether highly selective agents offer distinct therapeutic advantages. Based on current literature, the clinical efficacy and safety profile of upadacitinib appear largely comparable to those of pan-JAK inhibitors (16, 18). We report a case of successful treatment of CSVV in combination with upadacitinib. In contrast, there are several documented cases of CSVV being induced as an adverse effect by various biologic and targeted therapies. For instance, Mesut Tiglioglu et al. (6) reported a case of CSVV that emerged in a patient diagnosed with primary myelofibrosis during treatment with ruxolitinib. Xiaopeng Zhang et al. (19) described the onset of CSVV in a patient with Crohn's disease approximately 1 month after receiving a single injection of ustekinumab, which was administered due to inadequate response to conventional treatments. Similarly, Cury et al. (4) reported a case of CSVV that developed in a patient with follicular lymphoma after ten standard cycles of therapy with adalimumab.

As a highly selective JAK1 inhibitor, upadacitinib's pharmacological mechanism of action demonstrates a multi-faceted theoretical alignment with the key pathological processes of CSVV (20). In this case, the patient's favorable response to upadacitinib suggested a potential therapeutic role for JAK1 inhibition in managing refractory CSVV. The therapeutic effect observed may be explained by the drug's ability to interrupt key inflammatory pathways implicated in the pathogenesis of CSVV. As established, the central pathology involved immune complex deposition, complement activation, and subsequent neutrophil recruitment and activation (11). The release of pro-inflammatory cytokines such as IL-6 and Interferon-gamma (IFN-γ)from these activated immune cells further amplifies the inflammatory cascade (12, 13), and these cytokines signal predominantly through the JAK/STAT pathway (14). By selectively inhibiting JAK1, upadacitinib can block the signaling of these pivotal cytokines (21, 22). This action may mitigate neutrophil activation and the associated vessel wall damage by reducing downstream inflammatory mediators (23, 24). Additionally, inhibiting the JAK/STAT pathway may help stabilize endothelial function by modulating the expression of adhesion molecules (e.g., Intercellular Adhesion Molecule-1, Vascular Cell Adhesion Molecule-1) and factors influencing vascular permeability (25), thereby reducing leukocyte extravasation. While some CSVV cases are associated with autoantibodies, our patient's laboratory workup was negative for such markers. Nevertheless, the role of cytokines like IL-6 in B cell regulation suggests a broader potential mechanism for JAK inhibitors in immune-mediated vasculitis (26, 27). Therefore, the efficacy of upadacitinib in this case likely stems from its capacity to dampen the core inflammatory loop—driven by cytokines like IL-6 and IFN-γ–that sustains neutrophil-mediated vascular injury in CSVV.

Steroid-refractory CSVV occurs in patients whose vascular disease does not respond to standard therapy. Many different definitions can be found in the literature. Some articles define steroid-refractory CSVV as showing no improvement or worsening of disease activity within 4–6 weeks of standard treatment (28). In this case, the patient showed no response after 4 weeks of corticosteroid therapy, and the disease remained uncontrolled with new lesions appearing. After combination therapy with upadacitinib was initiated, the skin lesions gradually decreased, the patient's condition progressively improved, and the dosage of methylprednisolone was successfully tapered without recurrence of the rash. This suggested that upadacitinib likely had a positive therapeutic effect in this patient. Considering that during the treatment with upadacitinib, the patient continued to receive methylprednisolone and adjunctive medications, a potential auxiliary effect of these drugs on the patient's condition cannot be completely ruled out. This study has certain limitations, and future larger case series or randomized controlled trials are needed to clarify the efficacy of upadacitinib for CSVV.

## Conclusion

Currently, there is no standardized treatment regimen for refractory CSVV, and the use of conventional immunosuppressants carries a risk of significant adverse effects for patients. This article reports a successful case of CSVV, which was unresponsive to corticosteroid therapy, treated with upadacitinib. This outcome suggests that JAK inhibitors hold considerable promise as a future therapeutic avenue for vasculitic diseases. However, it is crucial to note that this report can only provide preliminary evidence based on a single case. Future research urgently requires larger case series and well-designed controlled clinical trials to definitively confirm the efficacy and safety of upadacitinib and similar agents for CSVV, thereby enriching the therapeutic options for this condition.

## Data Availability

The original contributions presented in the study are included in the article/supplementary material, further inquiries can be directed to the corresponding authors.
